# Retraction: The Polyherbal Wattana Formula Displays Anti-Amyloidogenic Properties by Increasing α-Secretase Activities

**DOI:** 10.1371/journal.pone.0328649

**Published:** 2025-07-21

**Authors:** The PLOS One Editors

After this article [1] was published, concerns were raised regarding results presented in Figs 2–4 and the statistical analyses reported in this study.

Specifically:

- The following panels appear similar despite representing different experimental results:
  - Fig 2A β-actin panel and Fig 3F β-actin panel lanes 1-4.
  - Fig 4B hβAPP WT panel and Fig 4B ADAM17^-/-^ panel with vertical flipping.
- The statistical analysis section of the Materials and methods reports that data were analyzed using unpaired t tests for pair wise comparisons. However, multiple experiments in this article [1] appear to present more than 2 variables, suggesting that statistical tests designed for the comparison of multiple variables should have been used instead.
- The underlying data are not provided with the article [1], contrary to the article’s Data Availability statement.

The corresponding author stated that there was an error in the preparation of Fig 2A β-actin panel, and that the Fig 3F β-actin panel was correct. They provided the underlying blot data for these panels, and stated that in Fig 2A in [1] all blots were spliced between lanes 3 and 4 to remove a lane corresponding to 50 µg/ml WNF. Upon editorial review of the underlying data, it was noted that the Fig 2A Mat βAPP Immat βAPP WNF 100 µg/ml lane in [1] corresponds to the removed 50 µg/ml WNF lane.

Regarding Fig 4, the corresponding author stated that an error occurred in the figure preparation. They stated that Fig 4A hβAPP WT and Fig 4B hβAPP WT panels are correct but that the Fig 4A hβAPP ADAM10^-/-^ and Fig 4B hβAPP ADAM17^-/-^ panels are incorrect.

Regarding the concerns for the statistical analyses reported in this article [1], the corresponding author stated that comparisons were made in pairs between each Wattana-treated condition and the control group independently. They stated that they did not conduct comparisons between the different Wattana-treated groups themselves and therefore unpaired t tests were used.

The corresponding author provided the individual-level underlying data for this article [1]. Upon editorial review, it was noted that the number of independent experiments reported in the figure legends for the western blot results are different to the number of values for each experimental group reported in the underlying individual-level data. The corresponding author stated that for the western blot experiments, the figure legends report the number of whole sets of independent experiments conducted. Within a blot, some experiments had more than one distinct control lane. Each control lane was compared individually to each treatment condition within a blot, without averaging the control group values within a blot. When more than one blot was run for the same independent samples all results from each blot were considered without averaging between blots. All treatment-control comparisons for all blots and all replicate blot results with the same samples were used to plot the graphs and perform the statistical analyses. The *PLOS One* Editors also noted that the sAPPα densitometry results in [1] were not normalized to a loading control.

In the underlying data, all individual values for the control groups in all western blot experiments in [1] were equal to 100 (% of control). However, the corresponding author provided a file underlying the statistical analyses for Fig 2A, showing that the values in the control group were not all equal to 100. The corresponding author stated that it is not possible to perform t tests when all values are equal to 100 for the control group.

The statistics reported in the article and the author’s explanations of the statistical approach and sample size were reviewed by an independent statistical expert. They commented that the statistical analyses in the article were insufficiently described. The use of unpaired t tests was not appropriate and a statistical test accounting for multiple comparisons should have been conducted. They stated that for the repeat controls within a blot, the controls should be averaged unless there is a scientific explanation for repeating the control-treatment comparisons. Regarding the control group values all equaling the same value, the statistical reviewer stated that non-zero variance is needed for all groups to conduct the unpaired t test as reported in the article, and they raised concerns for the validity of the statistical analyses reported in [1].

In light of the statistical review, the *PLOS One* Editors re-analyzed the western blot results using one-way ANOVA for the densitometry values without normalization to the control group and with averaging of the control lanes and technical blot replicates. The Editors noted that not all results reported as statistically significant in the article [1] were found to be significant upon re-analysis.

The *PLOS One* Editors retract this article in light of the statistical review, which calls into question the validity and reliability of the results and conclusions.

HHH and BV agreed with the retraction, stand by the article’s findings and apologize for the issues with the published article. SL, OT, NP, and PA agreed with the retraction and apologize for the issues with the published article. JFH, FC, and PG did not respond directly or could not be reached.
